# Comparative genome analysis of central nitrogen metabolism and its control by GlnR in the class Bacilli

**DOI:** 10.1186/1471-2164-13-191

**Published:** 2012-05-18

**Authors:** Tom Groot Kormelink, Eric Koenders, Yanick Hagemeijer, Lex Overmars, Roland J Siezen, Willem M de Vos, Christof Francke

**Affiliations:** 1 Kluyver Centre for Genomics of Industrial Fermentation, P.O. Box 5057, 2600 GA, Delft, The Netherlands; 2 TI Food and Nutrition, P.O. Box 557, 6700 AN, Wageningen, The Netherlands; 3 Laboratory of Microbiology, Wageningen University & Research Centre, Dreijenplein 10, 6700 HB, Wageningen, The Netherlands; 4 Netherlands Bioinformatics Centre, P.O. Box 9101, 6500 HB, Nijmegen, The Netherlands; 5 Centre for Molecular and Biomolecular Informatics, NCMLS, Radboud University Nijmegen Medical Centre, P.O. Box 9101, 6500 HB, Nijmegen, The Netherlands

## Abstract

**Background:**

The assimilation of nitrogen in bacteria is achieved through only a few metabolic conversions between alpha-ketoglutarate, glutamate and glutamine. The enzymes that catalyze these conversions are glutamine synthetase, glutaminase, glutamate dehydrogenase and glutamine alpha-ketoglutarate aminotransferase. In low-GC Gram-positive bacteria the transcriptional control over the levels of the related enzymes is mediated by four regulators: GlnR, TnrA, GltC and CodY. We have analyzed the genomes of all species belonging to the taxonomic families Bacillaceae, Listeriaceae, Staphylococcaceae, Lactobacillaceae, Leuconostocaceae and Streptococcaceae to determine the diversity in central nitrogen metabolism and reconstructed the regulation by GlnR.

**Results:**

Although we observed a substantial difference in the extent of central nitrogen metabolism in the various species, the basic GlnR regulon was remarkably constant and appeared not affected by the presence or absence of the other three main regulators. We found a conserved regulatory association of GlnR with glutamine synthetase (*glnRA* operon), and the transport of ammonium (*amtB-glnK*) and glutamine/glutamate (i.e. via *glnQHMP*, *glnPHQ*, *gltT*, *alsT*). In addition less-conserved associations were found with, for instance, glutamate dehydrogenase in Streptococcaceae, purine catabolism and the reduction of nitrite in Bacillaceae, and aspartate/asparagine deamination in Lactobacillaceae.

**Conclusions:**

Our analyses imply GlnR-mediated regulation in constraining the import of ammonia/amino-containing compounds and the production of intracellular ammonia under conditions of high nitrogen availability. Such a role fits with the intrinsic need for tight control of ammonia levels to limit futile cycling.

## Background

**Figure 1:**
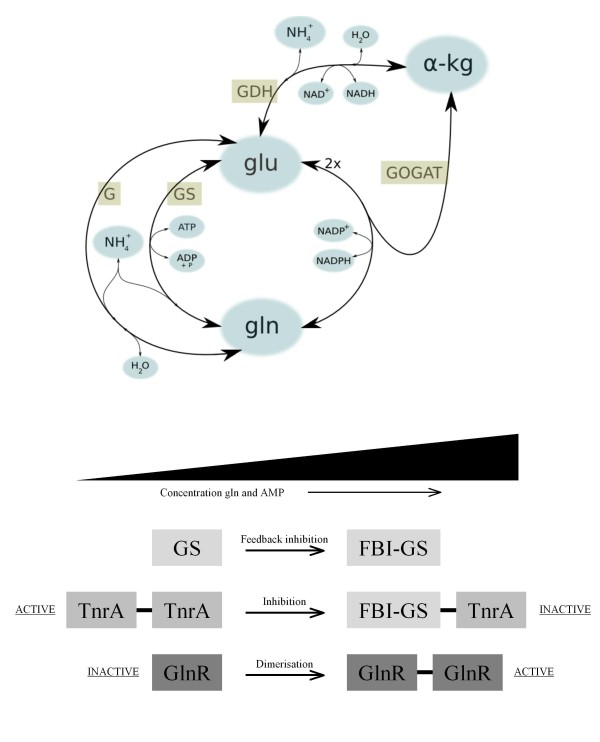
**The central reactions of cellular nitrogen metabolism and the related enzymes. (A) and the physiological range of regulator activity and associated feedback mechanisms (B)**. **A)** The metabolites are given in blue and the enzymes in green. Abbreviations: alpha-kg, alpha-ketoglutarate; glu, glutamate; gln, glutamine; GS, glutamine synthetase; GOGAT, glutamine alpha-ketoglutarate aminotransferase; GDH, glutamate dehydrogenase; G, glutaminase. **B)** Glutamine synthetase (GS) is feedback inhibited (FBI-GS) by glutamine and AMP. In the presence of FBI-GS, GlnR has a higher affinity for the binding site, whereas the activity of TnrA is repressed through a physical interaction with FBI-GS.

The assimilation and re-distribution of nitrogen within a cell is essentially controlled within the central metabolic conversions between alpha-ketoglutarate, glutamate and glutamine (Figure 1A). The enzymes that catalyze these conversions are glutamine synthetase (GS), glutaminase (G), glutamate dehydrogenase (GDH) and glutamine alpha-ketoglutarate aminotransferase (GOGAT). On a short timescale, the enzyme activity is controlled via activating and inhibitory molecular interactions. For instance, the activity of GS is suppressed via feedback inhibition (FBI-GS) by the product glutamine and by AMP [1]. Under conditions of nitrogen limitation a high GS activity is maintained to ensure a sufficient level of glutamine [2,3]. On a longer timescale, the enzyme levels are controlled via the activity of a limited number of transcription regulators.

Marked differences exist in the transcription control of the genes encoding the enzymes involved in central nitrogen metabolism across the bacterial kingdom. In the Gram-positive model organism *Bacillus subtilis*, the expression of these genes is mediated by four major transcription factors: CodY, GlnR, TnrA [4] and GltC [5,6]. Of these, GlnR, TnrA and GltC are specific for nitrogen metabolism whereas the global regulator CodY is linked to both carbon and nitrogen metabolism [7]. GltC is specifically associated with the control of the genes encoding glutamine alpha-ketoglutarate aminotransferase. The transcription factor GlnR is active during growth with excess nitrogen, whereas TnrA is active during nitrogen-limiting growth [4]. The change in activity of these transcription factors is affected directly by GS and feedback inhibition (FBI) of the enzyme (Figure 1B). In *B. subtilis* GlnR is activated in the presence of FBI-GS [8] and TnrA is inhibited through a physical interaction with FBI-GS [2,9]. It was also shown that TnrA binds to the PII-like regulatory protein GlnK, which is sensitive to ATP, Mg^2+^ and alpha-ketoglutarate [9,10]. The two proteins become tightly associated with the ammonium permease AmtB at a low level of ATP. In *Streptococcus mutans* cross-linking and pull-down assays demonstrated that GlnR also interacts with GlnK and that the interaction enhances the binding of GlnR to its cognate site upstream of the *glnRA* operon [11].

In *B. subtilis* and many other low-GC Gram-positives the genes encoding GlnR (*glnR*) and GS (*glnA*) constitute the operon *glnRA*[12]. In *B. subtilis* GlnR was reported to repress the transcription of the *glnRA* operon (negative autoregulation), and of *tnrA*[13] and the urease gene cluster (*ureABC*) [14]. On the other hand, TnrA was reported to affect the transcription of a larger set of genes/operons [15], for instance activating *glnQHMP* (encoding a glutamine ABC transport system [16]), *amtB-glnK* (i.e. *nrgBA*; encoding an ammonium permease and the regulatory protein GlnK [17]), *nasA* and *nasBC/DEF* (encoding proteins related to nitrite reduction [18]), *gabP* (encoding a gamma amino butyrate transporter [19]) and *pucR* (encoding the purine catabolism regulator [20]), while repressing *alsT* (encoding an H^+^/Na^+^ amino acid symporter [15]), *gltAD* (encoding glutamate synthase [21,22]) and *ilvBHC-leuABCD* (encoding branched-chain amino acid biosynthesis proteins [23]). Similarly, in the oral Streptococci *S. pneumonia* and *S. mutans* GlnR was reported to repress the transcription of the *glnRA* operon and of the *glnPQ* operon (encoding another glutamine ABC transport system) in both organisms and of *gdh* (encoding glutamate dehydrogenase) in the former, and the *amtB-glnK* and *citBZ-idh* operons (encoding aconitate hydratase, citrate synthase and isocitrate dehydrogenase [24]) in the latter organism [25,26].

Comparative genome analyses have shown that GlnR, TnrA and CodY are characteristic for the low-GC Gram-positive species although their distribution is not uniform. For instance, whereas GlnR is found in almost all *Bacillus* species, TnrA has been identified only in a few. It is an intriguing question whether in the absence of one of these main regulators the others take over its role. We therefore decided to extend (i.e. from 16 to 173 genomes) a previous comparative analysis reported by [27] to identify the presence of the regulators and the genes they regulate in the low GC Gram-positive species of the class Bacilli. This class includes the well-studied families Bacillaceae, Listeriaceae, Staphylococcaceae, Lactobacillaceae, Leuconostocaceae and Streptococcaceae.

We have redefined the binding motifs of GlnR and TnrA on basis of the available experimental and sequence data and used them to identify their respective regulons anew. For that purpose we have applied a footprinting approach formulated earlier by us [28] and a similar motif search procedure [29]. The difference in composition of the GlnR regulon was compared for the various taxonomic families within the class Bacilli and for species having only GlnR or also additional regulators. For most families we found a rather stable composition of the GlnR regulon and some species-specific connections, independent of the presence or absence of the other two regulators. The data imply that GlnR-mediated regulation serves predominantly to limit the import of ammonia/amino-containing compounds and, at the same time, to limit the production of intracellular ammonia.

## Results and discussion

### Presence/absence analysis of the genes encoding the central enzymes and regulators

We identified the orthologs of the genes encoding the enzymes of central nitrogen metabolism (G, GS, GDH and GOGAT), the related transport systems and the regulators CodY, GlnR and TnrA, in the sequenced genomes of species related to the class Bacilli on basis of BLAST searches with the sequences of experimentally verified proteins (see methods for details). In Tables 1 and 2 the analysis results for representative species of the orders Bacillales and Lactobacillales, respectively, are presented; the results for the complete set of analyzed species are given in Additional file 1. We observed a clear distinction in gene content between the two orders and between the different taxonomic families within the orders.

**Table 1:** Distribution and GlnR-mediated regulation of genes related to central nitrogen metabolism within the order Bacillales

NCBI PROJECT NAME \ GENES	*codY*	*tnrA*	*glnR*	*glnA*	*gdh*	*bdh*	*glutamin- ase (HA)*	*glutamin- ase (LA)*	*gltAB*	*gltA*^*BC*^	*gltS*	*amtB*	*glnK*	*glnQHMP*	*glnP*^*H*^*Q*	*glnT, alsT, yrbD, yflA*	*gltP, dctA, gltT, yhcL, nqt*
**A) order Bacillales**																	
*Anoxybacillus flavithermus WK1*	1	**1**	**1**	**2(2)**	2	1			1	1		**1**^**d**^			1	2(1)	1
*Geobacillus kaustophilus HTA426*	1	**1**	**1**	**2(1)**	2	2	1		1			**1**^**d**^		1	1	1	2
*G. thermodenitrificans NG80-2*	1	**1**	**1**	**2(2)**	2	2	1		1			**1**^**d**^			1	1	5(1)
*G. sp. Y412MC61*	1	**1**	**1**	**2(1)**	2	2	1		1			**2**^**d**^	**1**	1	1	1	2
*G. sp. C56-T3*	1	**1**	**1**	**2(1)**	2	1	1		1			**1**^**d**^		1	1	1	2
*G. sp. WCH70*	1	**1**	**1**	**1**	2	1	1		1	1				1		1	3
*Bacillus cereus ATCC 14579*	1		**1**	**1**^**b**^	1	1	1	1,0		1	1	**1**		1,2		6(1)	5(1)
*B. anthracis str. Ames*	1		**1**	**1**	1	1	1	1		1	1	**0,1**		1		6(1)	5(1)
*B. thuringiensis str. Al Hakam*	1		**1**	**1**	1	1	1	1		1	1	**1**		1		7(1)	6(1)
*B. weihenstephanensis KBAB4*	1		**1**	**1**	2	1	1			1	1	**1**		1		6(1)	5(1)
*B. atrophaeus 1942*	1	**1**	**1**	**1**	3	1	1	1	1		1	**1**	**1**	**1**		3(1)	4
*B. amyloliquefaciens DSM7*	1	**1**	**1**	**1**	2	1	1	1^c^	1		1	**1**	**1**	1		2(1)	3
*B. pumilus SAFR-032*	1	**1**	**1**	**1**	2	1	1	1	1		1	**1**	**1**	1		3	5(1)
*B. subtilis str. 168*	1	**1**	**1**	**1**	2	1	1	1	1		1	**1**	**1**	1,0		4(1)	4
*B. licheniformis ATCC 14580*	1	**1**	**1**	**1**	2(1)	1	1	1	1			**2(1)**	**2(1)**			6(1)	4
*B. megaterium DSM319*	1	**1**	**1**	**1**	3	1		1	1		1	**2(2)**	**2(2)**	1		3(1)	11(1)
*B. pseudofirmus OF4*	1	**1**	2(1)^e^	**2(2)**	4	1	1	1	1			**1**			1	3(1)	3
*B. clausii KSM-K16*	1	1		2	2	1	1		1			1			1	3	3
*B. halodurans C-125*	1	1		2	5	2	1	1	1			1				5	1
*B. selenitireducens MLS10*	1	1		1	4	1		1	1^b^	1		1			1	2	
*Lysinibacillus sphaericus C3-41*	1		**1**	**2(2)**^**c**^	2	1						**1**	**1**		1	3	5
*Oceanobacillus iheyensis HTE831*	1	1^a^	**1**	**1**	1	2		1	1		1	**1**	**1**		1	3	3
*Exiguobacterium sp. AT1b*	1	**1**	**1**	2(1)	1	1	1		1		1	**1**				6(1)	1
*Paenibacillus polymyxa E681*		**1**	**1**	2(1)	1		1		1			**1**^**d**^		2(1)	1	2	2
*Pb. sp. JDR-2*	1	**1**	**1**	**2(1)**	2	1	1		1		1	**3(1**^**d**^)	**1**	**1**			1
*Brevibacillus brevis NBRC 100599*	1		**1**	**2(2)**	2	1	1	1	1^b^		1	**1**^**d**^		1	1	1	3
*Ab. acidocaldarius DSM 446*	1		**1**	**3(1)**	2	1				1		**2(1)**	**1**				
*Listeria innocua Clip11262*	1		**1**	**1**	1				1			**1**	**1**		1		
*L. monocytogenes EGD-e*	1		**1**	**1**	1				1			**1**	**1**		1		
*L. seeligeri 1/2b str. SLCC3954*	1		**1**	**1**	1				1			**1**	**1**		1		
*Staphylococcus aureus Mu50*	1		**1**	**1**	1				1		1	**1**			**1**	2	2
*S. carnosus TM300*	1		**1**	2(1)	1				1		1					2	3
*S. epidermidis ATCC 12228*	1		**1**	**1**	1				1		1	**1**				1	2
*S. haemolyticus JCSC1435*	1		**1**	**1**	1				1		1	**1**				1	2
*S. lugdunensis HKU09-01*	1		**1**	**1**	1				1		1	**1**				1	2
*S. saprophyticus ATCC 15305*	1		**1**	**1**	2				1		1	**1**				2(1)	4
*Mc. caseolyticus JCSC5402*	1		**1**	**1**	1	1										2	3

The table summarizes the data retrieved for all species and strains which can be found in Additional file 1. The number of orthologs/homologs in every genome is indicated. For instance *Anoxybacillus flavithermus* WK1 contains 2 genes encoding glutamine synthetase. Every species is represented by one strain only. Species abbreviations: *Ab., Alicyclobacillus; B., Bacillus; G., Geobacillus; L., Listeria; Mc., Macrococcus; Pb., Paenibacillus; S., Staphylococcus.*

*Gene content.* The included genes encode the established regulators CodY, TnrA and GlnR [4]; the central enzymes Gln synthetase (*glnA*; [30]), Glu dehydrogenase (*gdh*; [31,32]), 3-hydroxybutyrate dehydrogenase (*bdh*[33,34]), high affinity (HA) and low affinity (LA) glutaminase, glutamate synthase (NADPH-dependent , large and small subunit *gltB* and *gltA*[21,22]; *B. cereus* type *gltA*; ferredoxin dependent *gltS*[35]), aspartate-ammonia ligase/asparagine synthetase (*asnA*[36] or *asnB*[37]), asparaginase (*ansA* or *ansZ*[38,39]); and the transport systems for ammonium (*amtB* with related regulator *glnK*; [40]), gln/glu using ATP (ABC-family, *glnP*^*H*^*Q* and *glnQHMP*) and glutamine-like proton symport (AGCS-family, *glnTalsTyrbDyflA*) and glutamate-like proton symport (DAACS-family, *gltPgltTyhclnqt*). In the latter two cases a variation in numbers was observed for different strains. In the other cases the numbers were far less variable (the ones that were variable are indicated as comma separated numbers).

*GlnR-mediated regulation.* Genes/operons that have a clear upstream GlnR binding site are marked by dark grey boxes (similarity score >87%), whereas genes/operons that are preceded by a less clear site are marked light grey (similarity score 80–87%). In case more genes are present encoding the same function then the number of genes with a clear binding site is indicated between brackets and if in addition the other gene(s) are preceded by a less clear site then the cell is marked light grey.

a) A GlnR-binding site is present but downstream of *tnrA* because the gene has opposite direction when compared to all other species.

b) For one sequence no ORF was called. However the gene could be identified using tBLASTN.

c) One of the sequences in two fragments.

d) Seems part of an operon that includes a gene with DUF294 domain and the gene *dnaQ*.

e) The *glnR* and *glnA* duplicates in *B. pseudofirmus OF4* are not part of a single operon and located at different positions on the genome.

**Table 2:** Distribution and GlnR-mediated regulation of genes related to central nitrogen metabolism within the order Lactobacillales

*NCBI PROJECT NAME \ GENES*	*codY*	*tnrA*	*glnR*	*glnA*	*gdh*	*bdh*	glutamin- ase (HA)	glutamin- ase (LA)	*gltAB*	*gltA*^BC^	*gltS*	*amtB*	*glnK*	*glnQHMP*	*glnP*^*H*^*Q*	*glnT*, *alsT*, *yrbD*, *yflA*	*gltP*, *dctA*, *gltT*, *yhcL*, *nqt*
**B) order Lactobacillales**																	
*Enterococcus faecalis V583*	1		**1**	**1**	1							1		**1**	**1**		3
*Lactobacillus acidophilus NCFM*				**1**								1		2			
*L. helveticus DPC 4571*				**1**								1		2			1
*L. crispatus ST1*				**1**				1						2			
*L. gasseri ATCC 33323*				**1**			1							2			1
*L. johnsonii FI9785*				**1**			1							2			1
*L. bulgaricus ATCC 11842*				**1**								1		3			
*L. fermentum IFO 3956*				**1**								1			1		1
*L. reuteri JCM 1112*				**1**			1	1				1		1	1		1
*L. brevis ATCC 367*			**1**	**1**								**1**		**1**			1
*L. plantarum WCFS1*			**1**	**1**	1							**1**		1	**1**		1
*L. salivarius UCC118*			**1**	**1**	1							**1**		**1**	**2(1)**^**e**^		1
*L. casei BL23*			**1**	**2(1)**	1,0				1			**1**		1	**1**^**b**^		
*L. rhamnosus GG*			**1**	**2(1)**	1				1			**2(1)**		1	**1**^**b**^		
*L. sakei 23 K*			**1**	**1**											1		2
*Pc. pentosaceus ATCC 25745*			**1**	**1**											**1**		1
*Leuconostoc citreum KM20*			**1**	**1**								**1**		**1**	**1**^**b**^		
*Ln. mesenteroides ATCC 8293*			**1**	**1**					1			**1**		**2**	**1**^**b**^		
*Oenococcus oeni PSU-1*			**1**	**1**								**1**		1	**1**^**d**^		
*Lactococcus lactis Il1403*	1^a^		**1**	**1**					1			**1**	**1**		**1**^**c**^		
*Streptococcus mutans UA159*	1		**1**	**1**	**1**				1			**1**	**1**	**1**	**1**^**c**^	1	
*S. equi*	1		**1**	**1**	1										**1**^**c**^	1	
*S. uberis 0140 J*	1		**1**	**1**	**1**									**1**	**1**^**c**^	1	
*S. gordonii str. Challis substr. CH1*	1		**1**	**1**	**1**										**1**^**c**^		1
*S. thermophilus CNRZ1066*	1		**1**	**1**	**1**							**1**	1	**1**	**1**^**c**^	1	
*S. sanguinis SK36*	1		**1**	**1**	**1**									**1**	**1**^**c**^	1	
*S. pneumoniae R6*	1		**1**	**1**	**1**									**1**	**1**^**c**^	1	
*S. mitis B6*	1		**1**	**1**	1							**1**	1	**1**	**1**^**c**^	1	
*S. agalactiae NEM316*	1		**1**	**1**	1							1		**1**	**1**^**c**^	1	
*S. pyogenes M1 GAS*	1		**1**	**1**											**1**^**c**^	1	
*S. suis BM407*	1		**1**	**1**	**1**									**1**	**1**^**c**^	1	

The table summarizes the data retrieved for all species and strains which can be found in Additional file 1. The number of orthologs/homologs in every genome is indicated. For *Gene content* and how *GlnR-mediated regulation* is indicated see the legend of Table 1. Every species is represented by one strain only. Species abbreviations: *L., Lactobacillus; Ln., Leuconostoc; Pc., Pediococcus; S., Streptococcus.*

a) *L. lactis* has a *codY* paralog named *codZ*[41].

b) There is a second *glnP*^*H*^ gene located next to the *glnP*^*H*^*,Q* operon.

c) The *glnP*^*H*^ gene encodes two H domains.

d) In *O. oeni* the gene *glnP*^*H*^ is in one operon with a gene encoding an asparaginase, whereas a *glnQ* homolog is missing.

e) In *L. salivarius* a second *glnP*^*H*^ gene is not part of an operon and is located elsewhere on the genome with respect to the *glnP*^*H*^*Q* operon.

Remarkably, within the family Lactobacillaceae, *Lactobacillus acidophilus* and its close relatives lack all three regulators. There are only three other species, *Bacillus halodurans*, *Bacillus clausii* and *Bacillus selenitrireducens* that lack a gene encoding GlnR. The global regulator CodY is present in most species except for those of the families Lactobacillaceae and Leuconostocaceae. TnrA is only present in species of the order Bacillales within the families Bacillaceae, Paenibacillaceae and the genus *Exiguobacterium* with the exception of the species and strains of the *Bacillus cereus* group, *Alicyclobacillus acidocaldarius*, *Brevibacillus brevis* and *Lysinibacillus sphaericus*.

Similarly, we observed a large variation in the presence of the enzymes of central nitrogen metabolism, but much less so in the related transport systems. The set of enzymes is complete within the family of the Bacillaceae and mostly reduced in the other families; in many of the Lactobacillaceae, Leuconostocaceae and Streptococcaceae only glutamine synthetase and one of the other enzymes is present. In the case of transport, at least one ammonium transporter AmtB (Amt-family; 1.A.11 in TCDB classification [42]), also referred to as NrgA [40], is present in most species, although the transporter is absent in more than half of the analyzed Streptococcaceae, in three *Bacillus anthracis* strains, in the gut-related Lactobacilli (e. g. *L. johnsonii* and *L. gasseri*) and in some meat-related species (e.g. *Lactobacillus sakei* and *Staphylococcus carnosus, Macrococcus caseolyticus*). It was recently put forward that transport of ammonia (NH_4_^+^) should be active and tightly regulated to limit futile cycling [43]. This control was suggested to be exerted by the small PII-like regulator GlnK, earlier referred to as NrgB (and as GlnB in e.g. *L. lactis*); the corresponding genes are indeed found genetically associated to *amtB* in many of the analyzed species. However, at the same time, it is absent in many others, including all analyzed Lactobacillaceae. Moreover, GlnK was shown to interact with TnrA in *B. subtilis*[9,10] and GlnR in *S. mutans*[11].

Most of the analyzed species carry one operon encoding a glutamine ABC transporter (PAAT-family; 3.A.1.3). Two variants of this system were identified: i) a *glnQHMP* operon found in *Escherichia coli* and *B. subtilis* and ii) a *glnP*^*H*^*Q* variant found in the Lactobacilli and Streptococci. This latter operon consists of 2 genes, in which *glnP*^*H*^ represents a fusion-gene of the permease *glnP* and the substrate-binding protein encoding *glnH*. In fact, in the case of the Streptococci the substrate-binding domain was duplicated to generate a *glnP*^*HH*^*Q* variant, whereas in some Lactobacilli the *glnP*^*H*^ gene has been duplicated. The *E. coli* system has been related to high-affinity glutamine transport [16], whereas the *L. lactis* system was shown to transport both glutamine and glutamate [44]. Recently, the *E. coli*-type system present in *Streptococcus mutans* was proven also to be involved in the transport of glutamate [45]. Remarkably, most of the species of the order Lactobacillales carry a copy of both types (Table 2). These Lactobacilli lack the genes encoding a glutamate dehydrogenase (*gdh*) or glutamate synthetase (*gltAB*). Therefore these species are unable to synthesize glutamate, which makes it essential to have a glutamate transport system.

Most of the analyzed species encode one or more transporters of the DAACS-family (2.A.13) and AGCS-family (2.A.25), with the exception of the species within the families Listeriaceae and Leuconostocaceae, some Lactobacillaceae and *L. lactis*. These transporter-protein families have been related to the cation symport of dicarboxylates and amino acids. The former family is represented by GltP (glutamate/aspartate [46]), GltT (glutamate [47]), DctA (C4-dicarboxylates including aspartate [48]), YhcL (or TcyP; cystine [49]) and Nqt (putative glutamate in *B. subtilis*), whereas the latter family is represented by GlnT (glutamine [50]), AlsT (amino acid [15]), YrbD (putative amino acid) and YflA (putative amino acid).

### Identification of a GlnR and TnrA specific binding motif

**Figure 2:**
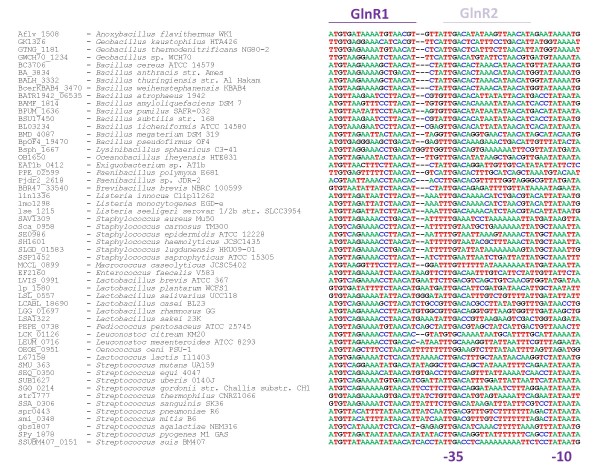
**Aligned upstream region of the*glnRA*operon in sequenced Bacilli.** The putative GlnR-binding sites and the-35 region (TTGAC) of the promoter are marked.

The protein sequences of GlnR and TnrA are highly similar and their reported DNA binding sites show little difference [27]. The palindromic consensus sequence has been defined as TGTNA-N7-TNACA [13,15,51-55]. Gel mobility shift assays indicated that TnrA and GlnR indeed bind to the same sites upstream of the *tnrA* gene and the *glnRA* operon, albeit with different specificity [13]. To achieve a separation of the predicted sites we have employed a genomic footprinting strategy that we formulated previously [28,56] to identify the GlnR-specific binding motif anew. The strategy involved the definition of Groups Of Orthologous Functional Equivalents (GOOFEs) on basis of conserved genomic context. Within these GOOFEs we assumed conservation of binding motif. In all analyzed species that contain *glnR*, the genetic association with *glnA* has been conserved. Moreover, for several species GlnR was shown experimentally to be autoregulatory and therefore the upstream region of the *glnRA* operon within all genomes was scanned for a conserved GlnR binding site. In line with earlier published observations we found a clear and strongly conserved binding site 3–6 nucleotides upstream of a putative-35 region (i.e. TTGAC) of the promoter in all analyzed species and a second binding site overlapping the promoter in many of the *Bacillus* species (Figure 2).

**Figure 3:**
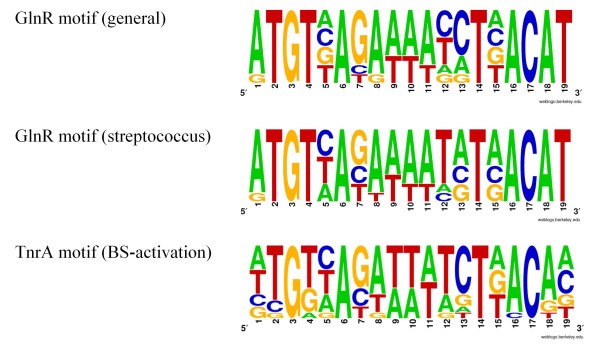
**Frequency representation of the putative GlnR and TnrA binding motifs. A)** GlnR-binding motif in species of the class Bacilli. **B)** GlnR-binding motif in species of the family Streptococci. **C)** TnrA-binding motif in *Bacillus subtilis.*

It was shown in a cross-regulation study that the binding site upstream of the promoter of the *glnRA* operon in *B. subtilis* is only involved in GlnR-mediated regulation [13]. Therefore, to tract potential differences between the GlnR and TnrA binding motifs, we used the conserved GlnR-binding sites upstream of the promoter to generate a family specific position frequency matrix (see methods). It appeared that the frequency representations of the motif varied slightly between the Streptococci and the other Bacilli (Figure 3A and B). Both motifs that were generated for GlnR adhered to the consensus motif [13,15,51-55] and were similar to the motif that was previously defined by [27]. Then, a TnrA-specific motif was created on basis of the TnrA sites upstream of *amtBansZgapPglnQnasAnasBnasDoppApucJpucRykzB* and *ywrD*. These binding sites were reported to relate to transcription activation in *B. subtilis* ([15,19,38,52,57-59] and raw data file 1) and are supposed to be TnrA-specific as GlnR has not been reported to activate transcription. The frequency representation of the TnrA-specific motif is given in Figure 3C. A comparison of the GlnR and TnrA specific motifs shows that there is limited difference. Yet the TnrA motif clearly lacks the conserved A and T at the 3′ and 5′ end, as was noted before. In fact, mutation of the conserved T at the 5′ end to a C or a G (but not A) was reported to abolish GlnR-mediated repression of the *glnRA* operon in *B. subtilis*[55], although [13] did not observe such an effect. Our new motifs also suggest that there is a slight preference for a G at position 7 and a C at position 13 which is less pronounced in the GlnR motif in Streptococci.

### The predicted GlnR and TnrA regulon of *B. subtilis*

The specific GlnR and TnrA motif were used to search the *B. subtilis* genome for similar sites using the Similar Motif Search (SMS) procedure described in the methods. The results of this search can be found in Additional file 2. Although the differences in GlnR and TnrA motif did not appear strong in first instance, the results of the motif search in *B. subtilis* suggest they are large enough to bring about some separation between GlnR and TnrA binding sites, in line with the observed variable affinities of these transcription factors for the same sites [13].

In principle the highest scoring sites are likely to be genuine binding sites and by using a relatively high cut-off score of 0.89 the majority (>70%) of experimentally validated sites was indeed captured for GlnR as well as for TnrA. Moreover, most other true binding sites scored just below the cut-off. Only 4 out of 22 reported TnrA binding sites were not recovered in this way. Some of the sites were actually found at a relatively large distance from the translation start (e.g. in the case of *ilvB*[60]) and many sites were found located in the shared regulatory region of neighboring genes located on opposite strands (so-called divergons). Although many sites were found in both searches, the similarity score was mostly clearly better for one than for the other. Genes/operons predicted to be controlled by both regulators included the known genes/operons *glnRA* (glutamine synthesis) and *tnrA*. Additional shared sites were found upstream of *alsTpucHpucJKLM* and the *amtB-glnK* operon. Although these sites have not been attributed to GlnR earlier and were described to be activated by TnrA [15,57], the relatively high simililarity score and the evolutionary conservation, also among organisms that lack TnrA, suggest they are true binding sites. In the case of the *amtB-glnK* operon (import of ammonia) it was formerly concluded that it is not repressed by GlnR on the basis of a singular observation. It was found that the *amtB-glnK* operon remained repressed in a GlnR deletion mutant (i.e. *glnR57*[12]) in the presence of glutamine, similar to the wild-type [61]. However this observation does not exclude repression by GlnR in case additional regulators are at play. In fact, in *L. lactis* it was shown that expression of the *amtB-glnK* operon is controlled by GlnR but also by CodY [62]. In *S. mutans* it was shown by electrophoretic mobility shift assay that GlnR binds to the promoter region of both the *glnRA* and *amtB-glnK* operon [11]. The same study identified GlnK as an activator of GlnR DNA-binding. Besides, the data in Tables 1 and 2 indicate that a putative GlnR-binding site upstream of *amtB* is present across almost all species of the class Bacilli. The conservation of these putative binding sites, including the conservation of the flanking A and T nucleotides (see Additional file 3), suggests GlnR represses the *amtB-glnK* operon in all analyzed species, and thus also in *B. subtilis*.

It was proposed that GlnR lacks the capability to recruit RNA polymerase and therefore acts solely as a repressor [13]. Given this lack of activating/recruiting capacity, it is to be expected that GlnR will only act on the expression of one gene in various divergons, like for instance on *tnrA* but not *ykzB*[58] and on *pucH* but not *pucR*[59]. Moreover, in various cases where TnrA was shown to activate transcription our analysis suggests the binding site is TnrA-specific, like for *gabPoppABCDF* and *glnQHMP*. Other genes/operons that seem to be TnrA-specific in *B. subtilis* include: *yclG*, encoding an uronase; *ydaB*, encoding an acyl-CoA ligase; *pel*, encoding a pectate lyase; *braB*, encoding a branced-chain amino acid-Na + symporter; and *pucABCDE*, encoding a xanthine dehydrogenase operon.

### The predicted GlnR regulon in oral Streptococci

GlnR binding-site predictions were performed for the oral Streptococci *S. pneumoniae* and *S. mutans* on basis of the Streptococci-specific motif (results in Additional file 4). For *S. pneumoniae D39* and *S. mutans UA159*, the genes/operons predicted to be controlled by GlnR were compared to the genes/operons whose transcription was most affected in a GlnR mutant [25,26]. We found good agreement between prediction and experiment for both organisms (see Table 3). In the case of *S. pneumoniae D39*, the most significantly up-regulated genes/operons, *glnP*^*HH*^*Q* and *gdh* were represented by the best hits in our analysis. The analysis also revealed the presence of a clear binding site in front of 2 other genes/operons, in line with the predictions of [27]. These included the second glutamine ABC transporter (*glnQHMP*) and an operon containing enzymes of the urea cycle (*arcAB*). The clear regulatory connection between GlnR and the *arcAB* operon (encoding arginine deiminase and ornithine carbamoyltransferase) was found in all sequenced *S. pneumoniae* strains, but was absent in the other species. The absence of a change in *arcAB* and *glnQHMP* expression upon inactivation of *glnR* may be explained by the presence of additional regulatory interactions.

**Table 3:** **Predicted GlnR regulon in*Streptococcus pneumoniae*(A) and*Streptococcus mutans*(B)**

gene/operon name	locus tag*	annotation	score (rank)	rank in exp.
A) *Streptococcus pneumoniae*				
*glnR,A*	SP_0501,0502	GlnR regulator, Glutamine synthetase	0.95 (1)	k.o.
*glnP*^*HH*^*,Q*	SP_1241,1242	Gln/Glu ABC transport system	0.92 (2)	1,2, SP_1243: 3
*gdh*	SP_1306	Glutamate dehydrogenase	0.90 (5)	4
*arcA,B*^a^	SP_2148,2150	Arginine deiminase, Ornithine carbamoyltransferase	0.91 (3) ^b^	-
*glnQ,H,M,P*^a^	SP_0610-0607	Gln/Glu ABC transport system	0.84 ^b^	-
B) *Streptococcus mutans (UA159)*				
*glnR,A*	Smu.363,364	GlnR, Glutamine synthetase	0.95 (1)	k.o.
*amtB,glnK*	Smu.1658,1657c	Ammonium transport system, GlnK regulator	0.95 (1)	1
*glnQ,H,M,P*	Smu.1519-1522	Gln/Glu ABC transport system	0.95 (2)	2
*citB,Z,idh*	Smu.670,671,672	Aconitate hydratase, Citrate synthase, Isocitrate dehydrogenase	0.95 (1)	3
*Smu.807*	Smu.807	Putative membrane protein	0.95 (1)	4
*glnP*^*HH*^*,Q*	Smu.806c,805c	Gln/Glu ABC transport system	0.95 (1)	5
*gdh*	Smu.913	Glutamate dehydrogenase	0.95 (1)	-
*Smu.68, thrC*	Smu.68,70	Hypothetical, Threonine synthase	0.92 (5)	-

The GlnR-binding site identifications were made as described in the methods for all strains with a published genome (data in Additional file 4). The composition of the regulon appeared identical between strains although the similarity scores of particular binding sites varied slightly. The table lists the numbers obtained with strains TIGR4 and UA159, respectively. Column 4: The ranking is based on the scores obtained with the similar motif search procedure, which provides various sites with identical scores and thus identical ranking. The absence of certain high scoring sites (e.g. ranked 4) was caused by the conservative criteria we applied for a site to qualify as a putative binding site. Column 5 gives the observed ranking on basis of the transcriptional response towards a *glnR* knock out mutation (k.o.) as derived from [25] and the ranking of operons that are downregulated in a *S. mutans glnR* knock out after exposure to acid stress for 30 minutes [26]. The ranking score was calculated by dividing the fold change in the *glnR* mutant by the fold change in the wild-type. Column 1 (a): In some strains the *arcA* and *glnQ* ORFs have not been called. The published sequence of strain R6 suggests *arcA* and *glnQ* are truncated in this strain. Column 4 (b): The composition of the binding site upstream of *arcAB* and glnQHMP varies between strains.

* Although [25] studied the effects in strain *D39*, the used microarrays were based on strains *TIGR4* and *R6*. For reasons of comparison we have therefore listed the locus tags in strain *TIGR4*.

In the case of *S. mutans UA159* the genes and operons found to be mostly affected in the knockout mutant [26] were the *nrgA-*SMU_1657c operon, coding for the ammonium transporter AmtB and its nitrogen regulatory protein GlnK; the *citB-citZ-idh* operon coding for aconitate hydratase, citrate synthase and isocitrate dehydrogenase; the *glnQHMP* and *glnP*^*HH*^*Q* operons encoding glutamine ABC transporters; and Smu.807*,* coding for a putative membrane protein, which is in a divergon with *glnP*^*HH*^Q. The best hits resulting from our analysis are also located upstream of the same operons. Moreover, we found a clear binding site preceding the genes *gdh* and *thrC*. The *citB-citZ-idh* operon has been shown to be essential for glutamate biosynthesis in *S. mutans*[24].

### Conserved genetic associations of GlnR and the effect of the other regulators

GlnR binding-site predictions were performed for selected genomes that represented all sequenced species of the class Bacilli. We then collected the function annotation of all proteins encoded by genes/operons downstream of a putative GlnR-binding site that fitted the selection criteria (see methods) to generate an overview of the regulatory connections that are conserved between more than three species (accumulated in Additional file 4). The results are summarized in Tables 1, 2 and 4. As expected, we found a conserved regulatory connection between GlnR and the *glnRA* operon in all analyzed species and with *tnrA* in all Bacilli. Only in a few species of the order Bacillales the related GlnR-binding sites deviated from the consensus (e.g. in some *Geobacillus* species). Another connection that was conserved in almost all of the analyzed species was that with *amtB* (often *amtB*-*glnK*).

**Table 4:** Distribution and function of additional genes-operons that are putatively linked to GlnR-mediated regulation

species	gene/operon	annotation (literature)
*A. flavithermus*, *G. C56-R3*, *G. kaustophilus*, *G. Y412MC61*, *L. seeligeri*, *P. JDR2*	*mcp*	regulation of chemotaxis
*B. megaterium*, *G. kaustophilus*, *G. Y412MC61*, *P. JDR2*, *P. polymyxa*	*pucR*	regulation of *puc* operon (purine catabolism) [20]
*B. amyloliquefaciens*, *B. atrophaeus*, *B. licheniformis*, *B. megaterium*, *B. pumilus*	*ycsFGI-kipIAR-ycsK*	includes regulation of sporulation [63]
*B. amyloliquefaciens*, *B. atrophaeus*, *L. salivarius*	*gabP*	transport of gamma-aminobutyrate [19]
*A. flavithermus*, *B. cereus*, *L. rhamnosus*, *O. oeni*, *P. JDR2*, *P. polymyxa*, *S. gordonii*, *S. sanguinis*	*opp*, *dpp*	transport of oligopeptide and/or dipeptide
*B. amyloliquefaciens*, *B. atrophaeus*, *B. megaterium* (2x), *B. subtilis*, *G. kaustophilus*, *G. Y412MC61*	*pucI*	transport of allantoin
*B. amyloliquefaciens*, *B. atrophaeus* (&*ywoB*), *B. subtilis* (&*ywoB*), *P. JDR2* (- *ywoD*)	*ywoCD*	transport (*ywoD*) and hydrolysis (*ywoC*)
*B. anthracis*, *B. cereus*, *B. thuringiensis*, *B. weihenstephanensis*, *P. JDR2* (*cpdB* only)	K01238<>*cpdB*	hydrolysis nucleoside 2′,3′-cyclic phosphate + H_2_O < = > nucleoside 3′-phosphate
*L. seeligeri*, *O. oeni*, *Pb JDR2*, *P. polymyxa*	*ansA,ansZ*	L-asparagine + H_2_O < = > L-aspartate + NH_3_[38]
*B. amyloliquefaciens*, *B. atrophaeus*, *B. licheniformis*, *B. pumilus*, *B. subtilis*	*argC*	N-Acetyl-L-glutamate 5-semialdehyde < = >
		N-Acetyl-L-glutamate 5-phosphate
*L. plantarum*, *L. rhamnosus*, *L. citreum*	*aspA*	L-aspartate < = > fumarate + NH_3_
*S. mutans*, *S. gordonii*, *S. sanguinis*, *S. suis*, *S. thermophilus*	*citZ-citB-idh*	conversion of citrate to alpha-ketoglutarate [24]
*B. amyloliquefaciens*, *B. atrophaeus*, *B. megaterium*, *B. pseudofirmus*, *G. C56-T3*, *G. kaustophilus*, *G. Y412MC61*, *P. sp. JDR2*	*nasDEF*	reduction of nitrite to NH_3_[18]
*B. atrophaeus*, *B. subtilis*, *G. Y412MC61*	*pucH*	allantoin + H2O < = > allantoate [20]
		[*pucF*: allantoate < = > ureidoglycolate + 2 NH3]
*B. anthracis* (*BC*), *B. cereus* (C), *B. thuringiensis* (*BC*), *B. weihenstephanensis* (C), *S. mutans* (*C*)	*thrBC*	L-homoserine + ATP < = > O-phosphohomoserine + ADP O-phosphohomoserine + H2O < = > threonine + P
		*ilvA*: threonine < = > 2-oxobutanoate + NH3] [24]
*B. amyloliquefaciens*, *B. megaterium*, *B. pseudofirmus*, *B. subtilis*, *P. JDR2*	*ureABC*	urea + H2O < = > CO2 + 2 NH3 [64]

The related data can be found in Additional file 4.

Various additional conserved connections were found, although these appeared far more family-specific. For instance, in the order Lactobacillales a genomic association with the genes of the two glutamine ABC transporter encoding variants *glnP*^*H*^*Q* or *glnQHMP* were identified, whereas this association appeared to be replaced by one with the sodium/proton amino acid symporters encoded by *gltT* (glutamate, [47]) and *alsT*[15] in various species within the family of the Bacillaceae. The AlsT protein is very similar to GlnT, a cation-glutamine symporter, i.e. showing a high degree of sequence conservation and having about the same length and the same number of predicted transmembrane helices. Although the protein is sometimes referred to as an alanine transporter, AlsT could well be a cation-glutamine or asparagine symporter.

Other associations that were conserved included that with the *gdh* gene (encoding glutamate dehydrogenase) and the *citBZ-icd* operon (encoding aconitase, citrate synthase and isocitrate dehydrogenase [24]) in many Streptococci, the *ureABC* operon (encoding urease [64]) and the *nasDEF* operon (encoding nitrite reductase [18]) in several *Bacillus* species and with several genes involved in purine and asparagine/aspartate transport and metabolism (e.g. *pucI* and *pucH* (encoding an allantoin transporter and allantoinase, respectively [20]), *ywoCD* (encoding an amidase and a transport protein), *ansA* and *ansZ* (both encoding asparaginase [38], *aspA* (encoding aspartase) and *asnA* (encoding aspartate-ammonia ligase).

We also found a clear GlnR-binding site upstream of several genes involved in regulation, for instance of *mcp* (chemotaxis, found in several *Geobacillus* species) and of the *ycsFGI-kipIAR-ycsK* operon (cellular development, found in several *Bacillus* species). In the initial description of the *ycsFGI-kipIAR-ycsK* operon [65], *ycsF* was related to the lactam (e.g. 2-pyrrolidinone) utilization gene *lamB* of *Aspergillus nidulans*[66], and *kipA* (orf12) was related to a urea amidolyase of yeast. Later, KipI was identified as a protein inhibitor of auto-phosphorylation of kinase A, the sensor histidine kinase responsible for processing post-exponential phase information and for providing phosphate input to the phosphorelay that activates developmental transcription via phosphorylated Spo0A, and KipA as a protein that counteracts the inhibition [63]. YcsG showed similarity to BraB (branched chain amino acid transport system II) of *Pseudomonas aeruginosa*[67]. The operon was found repressed upon growth on good nitrogen sources like ammonia and glutamine and derepressed on poor nitrogen sources [68], in line with repression mediated by GlnR. The association with the *ycsFGI-kipIAR-ycsK* operon connects GlnR-mediated regulation to the regulation of sporulation in some *Bacilli*.

Another important finding was that in *B. subtilis* many operons related to the purine degradation pathway are controlled by GlnR and/or TnrA, like *pucABCDEpucHpucIpucJKLM* and *ureABC*. The relation between purine catabolism and control by TnrA was established before experimentally. It was observed that a *tnrA* mutant strain could not use purines or its metabolic intermediates as a nitrogen source during nitrogen limited conditions [20]. Nevertheless, the extent to which both GlnR and TnrA are connected to the related operons is surprising.

We observed no clear dependency between the composition of the predicted GlnR regulon and the presence or absence of the other nitrogen-related regulators CodY and TnrA. For instance, there are only a few differences between the predicted GlnR regulon of *B. subtilis* and *B.cereus* suggesting that GlnR does not take over regulatory roles of TnrA. Similarly, the presence or absence of CodY does not seem to affect the size of the GlnR regulon in the Lactobacillaceae. In *L. lactis*, a species that has CodY, it was shown experimentally that at least three genes/operons (*amtBglnRA* and *glnP*^*HH*^*,Q*) are repressed by GlnR [62]. We indeed identified clear GlnR-binding sites in the upstream region of these three genes/operons in *L. lactis*. In *L. plantarum* and *L. monocytogenes,* two species that lack CodY, the same genes/operons appear to be preceded by a GlnR-binding site and only a few additional genes were found connected to GlnR indicating that GlnR does not take over the role of CodY in these species. The predicted GlnR regulon was smallest, consisting of only *glnRA*, in the meat isolates *Macrococcus caseolyticus* (a CodY and GlnR containing *Staphylococcus*) and *L. sakei* (a GlnR containing *Lactobacillus*).

## Conclusions

We have analyzed all sequenced Bacilli for the presence of genes encoding central nitrogen metabolism and transport of the related metabolites, and identified their connection to the nitrogen metabolism regulator GlnR. Although there is a considerable variety in the presence of the central enzymes GS, G, GDH and GOGAT, and in the number of available transport systems for the central nitrogen-related metabolites, the composition of the GlnR regulon is relatively invariable between species. Moreover, we hardly found an effect of the absence or presence of the other regulators CodY, TnrA and GltC on the size of the predicted GlnR regulon.

We made an initial conservative regulon prediction by restricting the regulatory association to those connections that are conserved between at least three species. In general, our findings are also in line with previous comparative in silico analysis performed on a limited number of species [27]. Careful redefinition of a specific GlnR-binding and a specific TnrA-binding motif caused a slight but clear separation in the predicted regulons. It is likely that the conserved A/Ts at the 3′ and 5′ end of the GlnR motif, which are absent in the TnrA motif, contribute significantly to the separation. For *B. subtilisS. pneumoniaeS. mutans* and *L. lactis* our predictions complied with the available experimental data. Moreover, within the Bacilli we identified several new potential members of the GlnR regulon, including the *ywoCD* operon and the *ycsFGI-kipIAR-ycsK* operon.

Our analysis confirmed that for most species the size of the GlnR regulon is relatively small. The main regulatory associations in the species of the class Bacilli are with the incorporation of ammonium into central metabolism (or with the production of ammonium at high glutamine concentrations!) via glutamine synthetase (*glnRA* operon), and with ammonium (*amtB-glnK*) and glutamine/glutamate transport (i.e. via *glnQHMP, glnPHQ, gltT, alsT*). At the same time, the lesser conserved associations point to a somewhat broader role. Many of the conserved associations include genes that are either directly (e.g. *ansAarcAaspAgdhnasDEFureABC*) or more indirectly (by controlling intermediate steps, e.g. *citBZ-idhpucHthrBC*) relate to the intracellular production of ammonia or are related to the import of aminated compounds (e.g. *gabPopp-dpppucIywoCD*). Thus, it appears that the main conserved role of GlnR is to prevent the influx and intracellular production of glutamine and ammonium under conditions of high nitrogen availability. The connection of GlnR-mediated repression with the control of intracellular ammonia concentration is interesting. Such a role fits with the intrinsic need for tight control of ammonia levels as put forward by [43], who argue that transport of ammonia (NH_4_^+^) should be tightly regulated to limit futile cycling by diffusion of ammonia out of the cell.

## Methods

### Data and tools

Complete genomic sequences and initial annotations were obtained from NCBI ([68]; June 2011). Multiple sequence alignments were made with ClustalX [69], and BioEdit [70] was used to analyze sequences and alignments. Specific bootstrapped neighbor-joining trees, with ‘correction for multiple substitutions’, were created using ClustalX and the trees were analyzed using LOFT [71] and Dendroscope [72]. The Microbial Genome Viewer 2.0 (http://mgv2.cmbi.ru.nl) was used to examine the function information within the genomic context. Frequency representations of aligned sequences were created with Weblogo [73]. Microarray data from *glnR* gene knockouts in *Streptococcus pneumoniae* and *Streptococcus mutans* used in this research were extracted from the Gene Expression Omnibus from NCBI [74].

The raw data resulting from the various analyses can be found at http://www.cmbi.ru.nl/bamics/supplementary/GrootKormelinketal_2012_GlnRregulon/. Data file 1: GlnR and TnrA motifs used for SMS; data file 2: GlnR motif search in *Bacilli* (w/o *Streptococci*); data file 3: GlnR motif search in *Streptococci*; data file 4: GlnR and TnrA motif search in *Bacillus subtilis*.

### Classification and annotation of protein sequences

To obtain all proteins of a certain family, a prominent representative was chosen (listed in the legend of Table 1) and a BLAST search [75] was performed (cut-off < e^−5^) on all publicly available sequenced Bacilli genomes. Then the list of collected sequences (given as Additional file 1) was inspected. For all enzymes, the sequences could be grouped into specific clusters based on BLAST e-value only. In practice we found a group of sequences with comparable (very) low e-values (<e^−30^) separated from the rest of the sequences with considerably higher e-values (separation < e^−15^). In the case of the transcription regulators the separation remained clear, however with higher e-values due to a short length of the regulator protein sequence. In the case of the transporters, for the Amt-family (1.A.11), the DAACS-family (2.A.13) and the AGCS-family (2.A.25) an e-value cut-off also sufficed to collect all family members, whereas for the PAAT-family; 3.A.1.3 (ABC transport) the coding sequences of the putative glutamine/glutamate substrate binding domains were aligned, the alignment was inspected by eye, deviant sequences were removed, and a bootstrap neighbour joining tree was generated (see [76]). The tree was divided into clusters on basis of the branching. For each cluster, single species representatives were considered orthologous. Genome context was used to verify the orthology/paralogy assignment and to provide additional information on the composition of the system.

### Motif definition and Similar Motif Scoring (SMS)

The upstream region of the conserved *glnRA* operon was retrieved for all species and the promoter region was aligned (see Figure 2). Then the conserved sequence upstream of the promoter was collected. For *B. subtilis* it was shown that this site is only involved in GlnR-mediated regulation [13]. The collection was used to generate a osition frequency scoring matrix for each taxonomic family (raw data file 1). It appeared that the frequency matrix was very similar for all species except for the Streptococci, where it was slightly different (illustrated in Figure 3). For the definition of a TnrA-specific binding site the upstream regions of genes whose transcription was shown to be activated by TnrA in *B. subtilis* (raw data file 1) were retrieved and the binding site was identified on basis of the published characteristic GlnR/TnrA motif and the short distance upstream of the promoter. The collection of sites was then used to generate a position frequency scoring matrix.

The position frequency matrices were used to identify potential binding sites in the analyzed genomes using a similarity search method formulated before by us [29]. The method relies on the fact that one of the most common practices observed in literature to reconcile prediction with experiment is to minimize the number of differences between the target and the query (or the ‘consensus’). In fact, this criterion can be captured in a straightforward scoring using only the position frequency matrix: Given any number of input sequences of size *i*, the nucleotide frequency *f*_N(j)_ (where N ∈ A,C,T,G; and frequency is in terms of fraction) at every position j = 1 to *i* can be used directly to provide all target sequences of size *i* with a score by just adding up the input-based frequencies that relate to the nucleotide composition of the target. Division of the score by the length of the sequence *i* results in a ‘similarity’ score that can range from 0 to 1. Dividing this number by the highest attainable score given the input matrix then yields a relative ‘similarity’ score. In case the input sequences are representative for high-affinity sites, the ranking of target sequences according to score should approximately correspond to a ranking based on affinity. The method was tested and appeared at least as good to identify putative regulatory elements on basis of known input motifs as the commonly used tool MAST [77], yet providing a similarity score that is far easier to interpret and use. We identified putative GlnR regulon members for all species on the basis of two simple criteria: i) a relative similarity score >87%; and ii) a position between 250 and 0 bases upstream of the predicted translation start. In some cases experimentally verified more distant sites were also included as well as known intergenic sites. The results are given in Additional file 4.

## Competing interests

The authors declare that they have no competing interests.

## Authors’ contributions

TGK conceived and designed the study, identified the GlnR and TnrA motifs and predicted their regulons, and drafted and revised the manuscript. EK helped designing the study and performed motif searches. YH implemented and validated the motif search algorithm. LO helped designing the study and revising the manuscript. RJS and WMV coordinated the study and helped revising the manuscript. CF coordinated the study and helped designing it, carried out the classification and annotation of protein sequences and helped drafting and revising the manuscript. All authors read and approved the final manuscript.

## Supplementary Material

Additional file 1 Distribution of genes related to central nitrogen metabolism. Click here for file

Additional file 2 *Bacillus subtilis* GlnR and TnrA regulon. Click here for file

Additional file 3 GlnR motif search in *amtB* upstream regions. Click here for file

Additional file 4 Conserved GlnR-mediated regulation. Click here for file
